# Transcriptomic and Functional Approaches Unveil the Role of tmRNA in Zinc Acetate Mediated Levofloxacin Sensitivity in Helicobacter pylori

**DOI:** 10.1128/spectrum.01152-22

**Published:** 2022-11-10

**Authors:** Hongjin Tao, Fansen Meng, Yu Zhou, Jiao Fan, Jing Liu, Yingjie Han, Benjamin B. Sun, Gangshi Wang

**Affiliations:** a Department of Gastroenterology, The Second Medical Center & National Clinical Research Center for Geriatric Diseases, Chinese PLA General Hospital, Beijing, People’s Republic of China; b Medical School of Chinese PLA, Beijing, People’s Republic of China; c Department of Laboratory Medicine, Second Medical Center, Chinese PLA General Hospital, Beijing, People’s Republic of China; d Institute of Geriatrics, National Clinical Research Center of Geriatrics Disease, Second Medical Center, Chinese PLA General Hospital, Beijing, People’s Republic of China; e Department of Oncology, Fifth Medical Center, Chinese PLA General Hospital, Beijing, People’s Republic of China; f MRC/BHF Cardiovascular Epidemiology Unit, Department of Public Health and Primary Care, University of Cambridge, Cambridge, United Kingdom; Vanderbilt University Medical Center

**Keywords:** Helicobacter pylori, zinc acetate, levofloxacin resistance, transcriptomics, *ssrA*

## Abstract

Rapid increase in resistance of Helicobacter pylori (H. pylori) has hindered antibiotics-based eradication efforts worldwide and raises the need for additional approaches. Here, we investigate the role of zinc-based compounds in inhibiting H. pylori growth and modulating antibiotic sensitivities, interrogate their downstream transcriptomic changes, and highlight the potential mechanism driving the observed effects. We showed that zinc acetate inhibited H. pylori growth and increased H. pylori sensitivity to levofloxacin. Transcriptomic profiling showed distinct gene expression patterns between zinc acetate treated groups *versus* controls. In particular, we independently replicated the association between zinc acetate treatment and increased *ssrA* expression. Knockdown of *ssrA* restored levofloxacin resistance to levels of the control group. In this study, we first demonstrated the role of zinc acetate in H. pylori growth and antibiotic sensitivities. Additionally, we explored the transcriptomic perturbations of zinc acetate followed by functional knockdown follow-up of differentially expressed *ssrA*, highlighting the role of tmRNA and trans-translation in H. pylori levofloxacin resistance. Our results provide alternative and complementary strategies for H. pylori treatment and shed light on the underlying mechanisms driving these effects.

**IMPORTANCE** Helicobacter pylori (H. pylori) eradication plays an important role in gastric cancer prevention, but the antimicrobial resistance of H. pylori is fast becoming a growing concern. In this study, we investigated the role of zinc acetate in inhibiting H. pylori growth and modulating antibiotic sensitivities *in vitro*. Additionally, we explored the transcriptomic perturbations of zinc acetate followed by functional knockdown follow-up of differentially expressed *ssrA*, highlighting the role of tmRNA and trans-translation in H. pylori levofloxacin resistance. Our results open up a new horizon for the treatment of antibiotic-resistant H. pylori.

## INTRODUCTION

Helicobacter pylori (H. pylori) infection is one of the leading causes of chronic gastric inflammation, predisposing individuals to peptic ulcerations and gastric cancer (1–3). Approximately more than half of the worldwide population carry H. pylori within the gastric mucosa with prevalence in certain regions such as Asia exceeding 80% (4, 5). Eradication of H. pylori reduces the long-term risks of peptic ulcers and gastric cancer (6, 7) and is fast becoming an emerging global health issue (8).

Current first-line treatment for H. pylori in general consists of a proton pump inhibitor with a combination of three or four antibiotics, such as penicillin-based or fluroquinolones combined with macrolides and metronidazole, depending on local resistance patterns (9, 10). The combination of antibiotic treatment reflects the challenges in H. pylori eradication (11), and rapidly increasing resistance to current antibiotic therapies (12) is a particular cause for concern. Therefore, additional means to inhibit H. pylori growth and increase their sensitivity to existing antibiotics are needed to tackle this increasingly difficult-to-treat endemic.

Various alternate treatment approaches have been investigated previously (reviewed in detail in [13]); however, many showed limited efficacy. Metal complexes, such as bismuth, are commonly added to H. pylori combination therapies in widespread clinical settings (14). Zinc-based complexes, in particular, such as the addition of polaprezinc (a chelate compound of zinc and l-carnosine) to combination therapies, have been shown to improve H. pylori eradication (15, 16). Currently, there have been limited studies examining effects of zinc acetate on H. pylori growth and sensitivities to antibiotics.

Here, we demonstrate (i) the inhibitory effect of zinc acetate on H. pylori growth *in vitro*, (ii) explore the transcriptomic changes in response to zinc acetate, and (iii) investigate the knockdown effects of the differentially expressed transfer-messenger RNA (*ssrA*) on antibiotic sensitivity.

## RESULTS

### Zinc acetate inhibits H. pylori growth.

**FIG 1:**
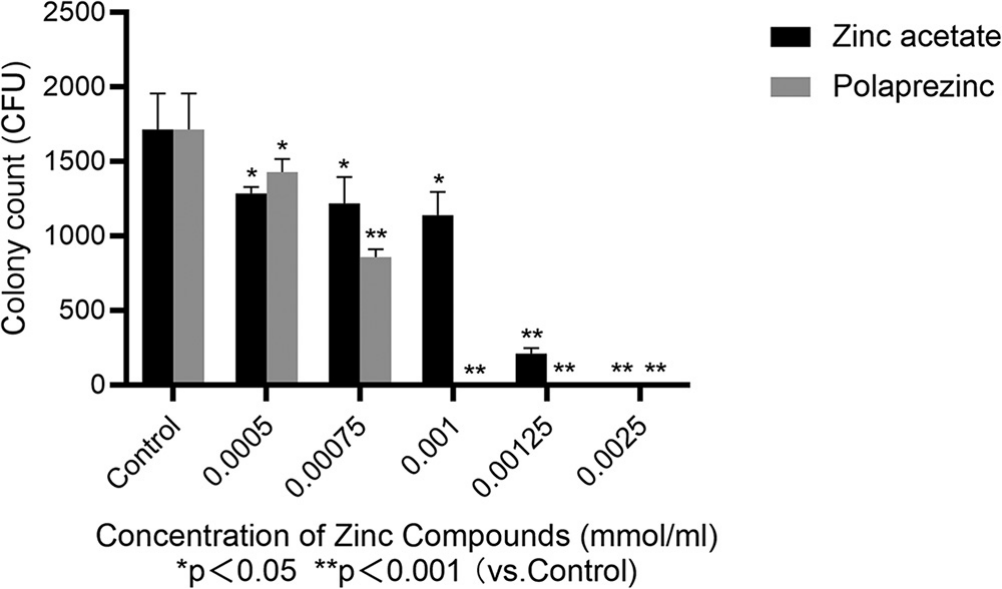
Zinc compounds inhibit the growth of H. pylori (NCTC 11637).

We cultured H. pylori (NCTC 11637) with zinc acetate for 3 days at 37°C. H. pylori growth was inhibited at increasing concentrations of zinc acetate, which becomes apparent at 0.00125 mmol/mL, with complete inhibition observed at 0.0025 mmol/mL (*P* < 0.001, versus control). Similar effects were also observed with polaprezinc (Fig. 1). In addition to the standard strains, we also tested the resistance of various clinical H. pylori strains to zinc acetate (and polaprezinc) and found broadly comparable MICs across the various strains (Table S1).

### Zinc acetate increases H. pylori sensitivity to levofloxacin.

Next, we investigated changes in H. pylori (NCTC 11637) sensitivity to levofloxacin, clarithromycin, and tetracycline after culturing with zinc acetate or polaprezinc for 3 days. MICs of levofloxacin and clarithromycin decreased after H. pylori exposure to both zinc acetate and polaprezinc, although only the MIC of levofloxacin strictly remained below the resistance breakpoint (https://www.eucast.org/clinical_breakpoints/) after exposure to zinc acetate (Table 1, Fig. S1). MICs of tetracycline remained unchanged in both conditions.

**TABLE 1:** Zinc compounds affect the MIC (μg/mL) of antibiotics to H. pylori NCTC 11637^*a*^

Antibiotics	Control	Polaprezinc	Zinc acetate
Levofloxacin	1–1.5	0.75–1	0.5–0.75
Clarithromycin	0.5–1	0.25–0.5	0.25–0.5
Tetracycline	0.5–1	0.5–1	0.5–1

a Breakpoints of antibiotic sensitivity: Levofloxacin: MIC ≤ 1 μg/mL; Clarithromycin: MIC ≤ 0.25 μg/mL; Tetracycline: MIC ≤ 1 μg/mL.

### Transcriptomic changes in response to zinc acetate.

**FIG 2:**
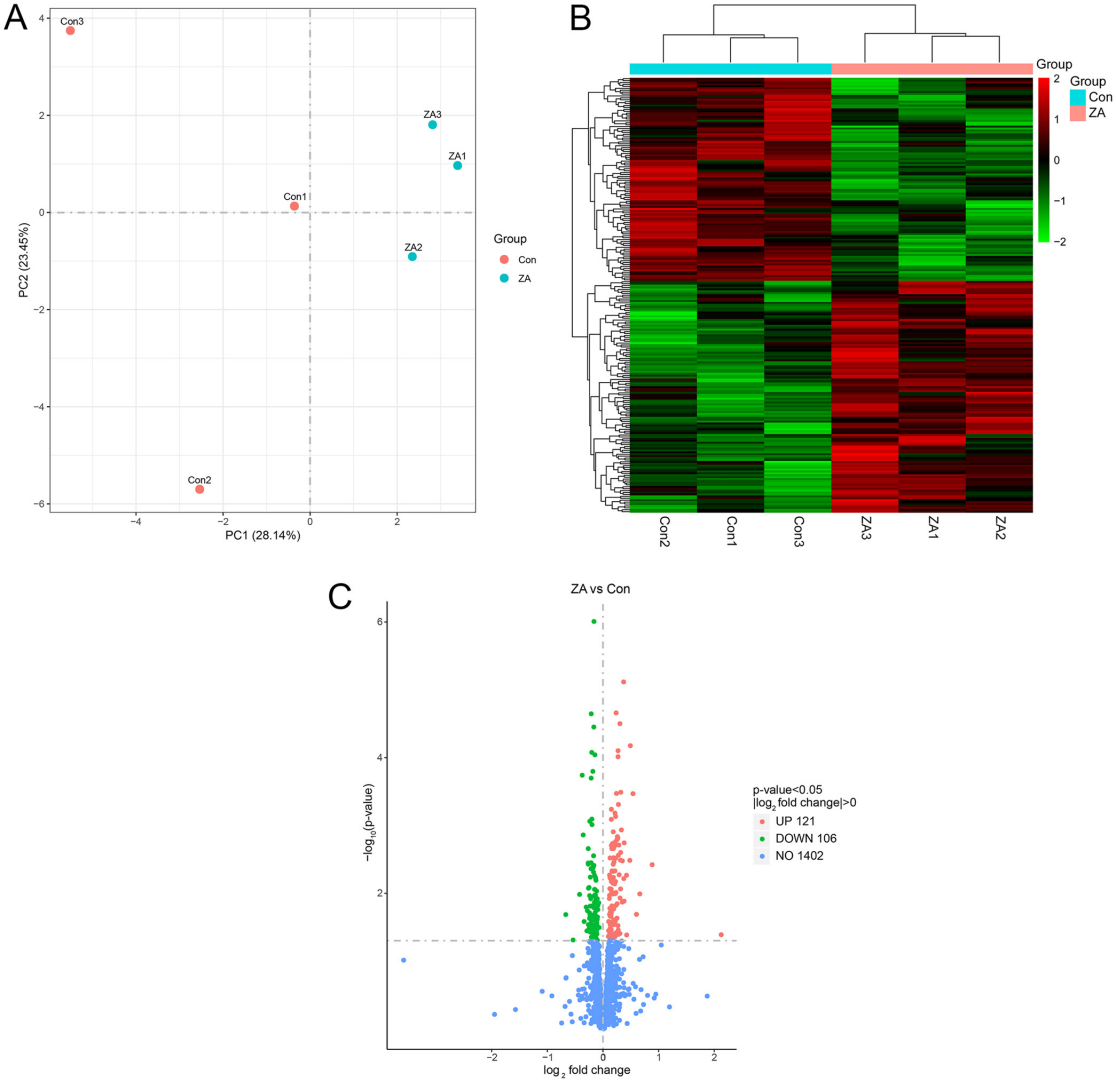
Differentially expressed genes (DEGs) identified by transcriptomic (RNA) sequencing. (A) Principal-component analysis (PCA) analysis of tested samples. (Con, control group; ZA, zinc acetate treated group). (B) Hierarchical clustered heatmap of differentially expressed genes (*n* = 227) between control and treated group. (C) Volcano plot exhibition of DEGs. The vertical axis represents the -log_10_ (*P*-value), the horizontal axis displays the log_2_ fold change. Red points signify the upregulated transcripts (*n* = 121), green points stand for downregulated transcripts (*n* = 106). Vertical dashed lines indicate absolute log_2_ fold change = 0 and horizontal dashed lines indicate *P*-value = 0.05.

To investigate the transcriptomic changes associated with decreased resistance of H. pylori to levofloxacin after exposure to zinc acetate, we used RNA-sequencing to assess gene expression changes after culturing H. pylori with zinc acetate for 3 days *versus* controls (*n* = 3 each). The zinc acetate treated samples were separable from the control groups based on principal-component analysis (PCA) using the transcriptomic profiles (Fig. 2a) and displayed distinct gene expression profiles (Fig. 2b). In total, we found 227 nominally significant (*P < *0.05) differentially expressed genes (DEGs) between the treated and control groups, with 23 DEGs significant after adjustment for multiple correction (Fig. 2c, Table S2). Additionally, we tested whether DEGs were enriched for any GO processes or KEGG pathway, and we did not find any significant enrichment after multiple correction (Table S3, 4). Genome sequencing was also performed on H. pylori NCTC 11637 with zinc acetate treatment versus controls (*n* = 3 each). Comparative genomics analyses, including indels, single nucleotide polymorphisms, structural variations, synteny analyses, did not reveal changes as a result of zinc acetate exposure (Table S5).

**FIG 3:**
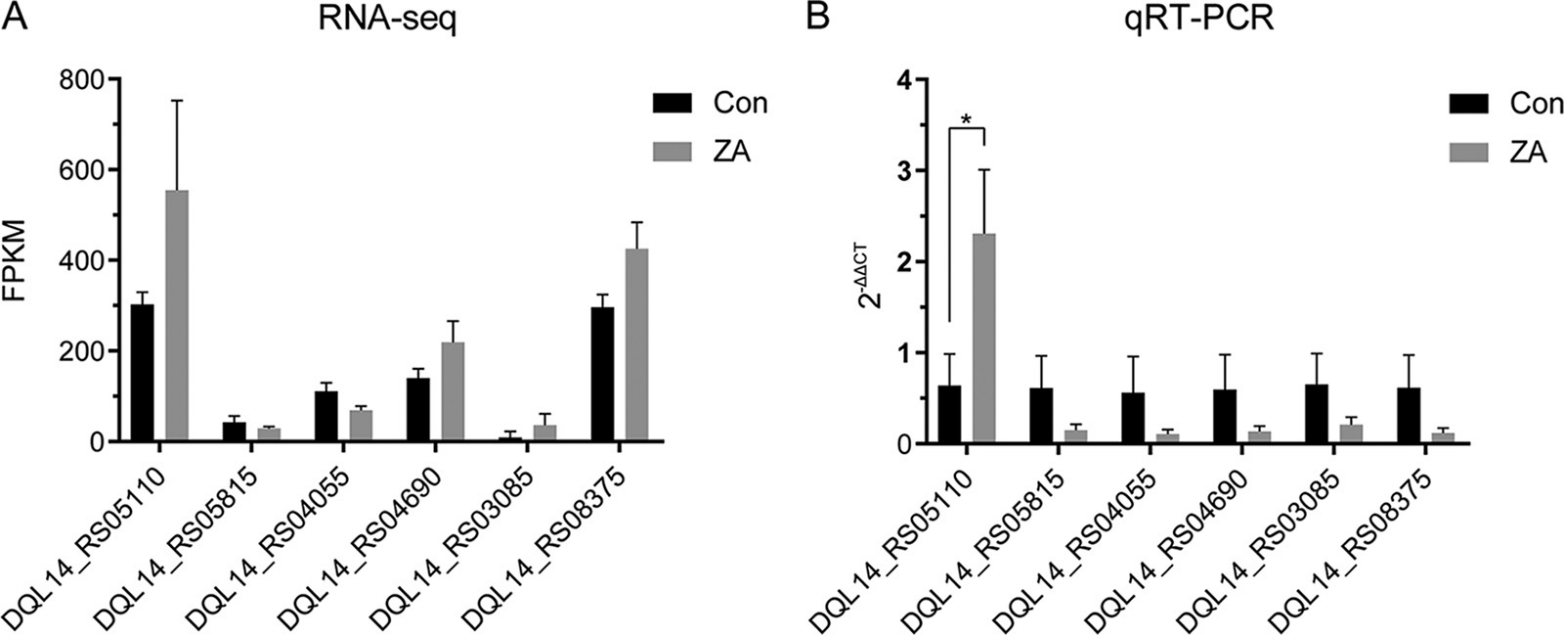
Validation of selected candidate genes using qRT-PCR. (A) Expression of 6 selected genes measured by RNA sequencing and assessed by Fragments Per Kilobase of exon model per Million mapped fragments (FPKM). Error bars indicate standard deviation. (B) Expression of 6 selected genes measured by qRT-PCR. 2^-ΔΔCt^ method was applied to evaluate gene expression differences. Error bars indicate standard deviation. Con, control group; ZA, zinc acetate treated group. (* *P* < 0.05).

We independently tested 6 DEGs with the largest absolute fold expression changes (Table 2, Fig. 3a) that were nominally significant using qRT-PCR (*n* = 3). We observed significant and consistent changes in expression for *ssrA* (DQL14_RS05110, also known as transfer- and messenger-RNA, tmRNA), where expression increased by 2.6-fold (*P = *0.02, Fig. 3b) after incubation with zinc acetate.

**TABLE 2:** Six selected differentially expressed genes measured by qRT-PCR

Gene ID	Gene name	Gene description	ZA versus Con Log_2_ (Fold Change)	ZA versus Con *P* value
DQL14_RS05110	ssrA	Transfer-messenger RNA	0.874	3.81E-03
DQL14_RS05815	hopJ	Hop family outer membrane protein HopJ/HopK PF01856:Helicobacter outer membrane protein	–0.548	4.89E-02
DQL14_RS04055		Hypothetical protein	–0.679	2.06E-02
DQL14_RS04690		Hypothetical protein	0.652	1.03E-02
DQL14_RS03085	rpmG	50S ribosomal protein L33	2.116	4.11E-02
DQL14_RS08375		Cag pathogenicity island protein	0.530	3.40E-04

### Acidity affects the transcription of *ssrA*.

The pH of liquid cultures was adjusted by the addition of HCl to pH 6.0, 4.5 and 3.0 with 5 mM urea. After NCTC 11637 was incubated in acidity gradient liquid media for 4 h, the *ssrA* gene expression was evaluated by qRT-PCR. Compared with the control group (pH 7.4), NCTC 11637 incubated in acidic media (pH 4.5 and 3.0) has a significantly increased *ssrA* expression to 3.9 times (*P < *0.05, Fig. S2).

### Knockdown of *ssrA* restores H. pylori resistance to levofloxacin.

**FIG 4:**
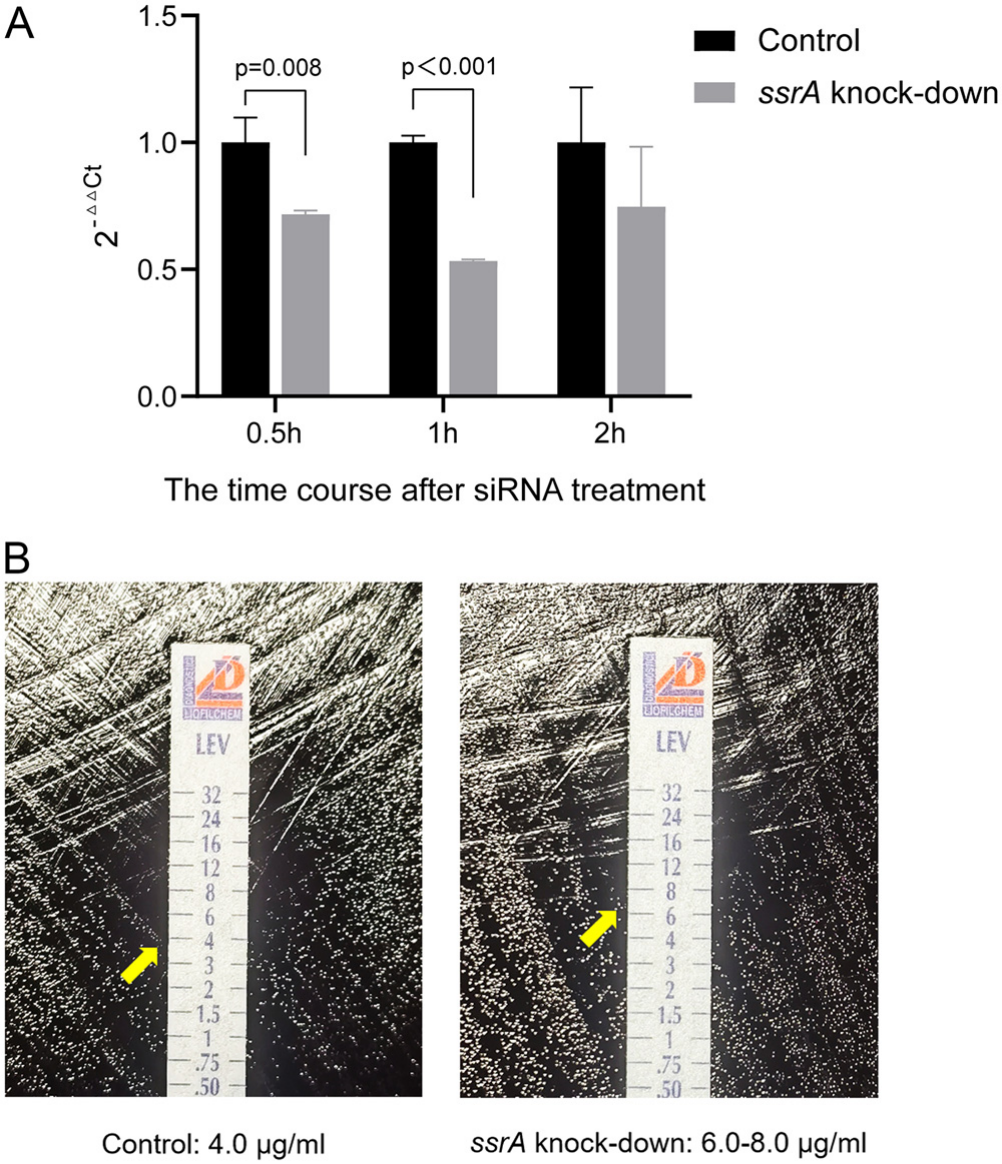
(A) The levels of *ssrA* expression after knockdown evaluated by qRT-PCR. 2^-ΔΔCt^ was used to evaluate the difference of gene expression (*P*-value by independent samples *t* test). (B) The impact of *ssrA* knockdown on levofloxacin sensitivity. Left: H. pylori electroporation at 2500 V for 5 ms as control; the MIC of levofloxacin was 4.0 μg/mL. Right: H. pylori electroporation at 2500 V for 5 ms and transformed by siRNA to *ssrA* for 1 h; the MIC of levofloxacin was 6.0–8.0 μg/mL.

After we replicated and validated increased expression of *ssrA* after zinc acetate treatment, we hypothesized whether *ssrA* expression is a potential causal mechanism driving the observed zinc acetate mediated H. pylori sensitivity to levofloxacin. First, we performed *ssrA* knockout in H. pylori, which led to complete inhibition of H. pylori growth, suggesting *ssrA* is essential for H. pylori growth (Fig. S3). Subsequently, we performed knockdown of *ssrA* in H. pylori, whereby siRNA *ssrA* knockdown reduced *ssrA* expression by 46%. The time course of knockdown results after *ssrA* siRNA treatment indicated that *ssrA* mRNA expression decreased the most after 1 h (Fig. 4a). MICs of levofloxacin for the zinc acetate treated group after *ssrA* knockdown were restored to levels similar to controls, consistent with *ssrA* being a driver of zinc acetate associated levofloxacin sensitivity (Fig. 4b).

## DISCUSSION

Rising antibiotic resistance to H. pylori is fast becoming a widespread global health concern, driving the need for alternative and complementary treatment approaches. Here, in this study, we demonstrated the role of zinc acetate in inhibiting H. pylori growth and increasing the sensitivity of H. pylori to levofloxacin, a commonly used antibiotic in H. pylori treatment. In the process, we also reproduced previously observed effects of polaprezinc on H. pylori growth (16).

This is one of the first studies to examine the transcriptomic perturbations associated with zinc acetate treatment, which showed distinct gene expression profiles compared to controls. Widespread transcriptomic perturbations were also previously seen after changes in environmental conditions such as temperature (17, 18). This suggests significant transcriptomic changes occur in H. pylori in response to biophysical as well as chemical perturbations. While we did not find evidence of enrichment for any specific pathways, we were able to test and validate the effects of the specific gene *ssrA* in detail via hypothesis-driven functional follow-up.

Through siRNA knockdown of *ssrA*, we were able to restore H. pylori resistance to zinc acetate induced levofloxacin sensitivity, suggesting an important role of *ssrA* in driving these observed effects. *SsrA* codes for the highly conserved tmRNA, a specialized RNA with properties of both a tRNA and an mRNA. tmRNA is one of the key components of trans-translation, a ubiquitous bacterial mechanism that resolves ribosome stalling caused by nonstop mRNAs, which is diversely implicated in many processes such as responding to stress, pathogenesis, and differentiation (19). Trans-translation is an essential function in bacteria (20), and plays regulatory roles in organisms such as H. pylori (21), Escherichia coli (22), Streptomyces coelicolor (23), etc. We showed that complete *ssrA* knockout inhibits H. pylori growth completely, suggesting tmRNA system is essential for H. pylori growth, consistent with similar reports in other bacterial species (24). Previous literature suggests that deletion of the tmRNA system reduces trans-translation and results in increased susceptibility to oxidative stress and antibiotics that inhibit protein synthesis and transcription (25). However, we demonstrated that zinc acetate treatment led to increased *ssrA* expression and sensitivity to levofloxacin and knockdown of *ssrA* led restored resistance. Our results are consistent with Streptococcus pneumoniae containing *ssrA* deletion becoming more resistant to levofloxacin and other fluoroquinolones (25). The exact mechanism by which this effect occurs in H. pylori remains to be elucidated.

Our study revealed the effect of zinc acetate on *ssrA* gene expression in H. pylori *in vitro*. However, we have not explored the changes in the bacterial transcriptome *in vivo* condition. Previous studies found that the gastric acid environment *in vivo* was pH 1.0–6.0 (26, 27), whereas acidic conditions influenced H. pylori on the transcription of different genes related to adaptation to acidity, antioxidant response, mobility, adherence, and pathogenicity (T4SS) (28). What’s more, acidity influenced the expression of genes related to the bactericidal effect of antibiotics (29). We investigated the transcriptional change on *ssrA* under acidic conditions and found *ssrA* was significantly upregulated at pH 4.5 and pH 3.0. This implied that the transcription of *ssrA* may also be upregulated in the gastric environment *in vivo*.

In summary, our results suggest that zinc acetate reduces H. pylori growth and increases sensitivity to levofloxacin through upregulating tmRNA system along with other distinct transcriptomic changes and modulating the trans-translational system. Our findings support zinc acetate as a useful adjunct in fluoroquinolone-based antibiotic therapies against resistant H. pylori.

## MATERIALS AND METHODS

### Bacteria strains and culture conditions.

H. pylori NCTC 11637, Sydney strain 1 (SS1), and clinical strains used in this study have been identified using 16S rRNA gene sequencing. H. pylori strains were cultured under microaerophilic condition (5% O_2_, 10% CO_2_, and 85% N_2_) on solid culture media composed of Karmali Agar Base (CM0935, Oxoid, UK) and 5% sterile defibrinated sheep blood (XLF Medical Sales Co., Beijing, China). Liquid culture medium was brain heart infusion (BHI) supplemented with 10% fetal calf serum (FBS), 0.25% yeast extract, and selective supplement (SR0147E, Oxoid, UK).

### Bacteria growth at different concentrations of zinc compounds.

Culture mediums containing different concentrations (0, 0.0005 mmol/mL, 0.00075 mmol/mL, 0.001 mmol/mL, 0.00125 mmol/mL, 0.0025 mmol/mL) of zinc acetate or polaprezinc (Broadwell Pharmaceutical, China) were prepared. H. pylori NCTC 11637 was resuspended to 10^5^ CFU/mL, and 100 μL of bacteria suspension was evenly coated on culture medium and colonies were counted after incubating at 37°C for 3 days.

### MICs of zinc compounds to H. pylori.

H. pylori strains—two standard strains (NCTC 11637 and SS1) and eight clinical strains (19004, 20050, 27054, 28038, 827, L1, L2, L3)—were resuspended in sterile PBS to 2 McFarland. One hundred μL bacteria suspension was evenly coated on culture medium with 0.0005 mmol/mL, 0.001 mmol/mL, 0.0025 mmol/mL, 0.005 mmol/mL, 0.01 mmol/mL of zinc compounds. After incubating at 37°C for 3 days, concentration of the zinc compounds in the plate with no bacterial growth was regarded as MIC.

### Zinc compound effects on MICs of antibiotics to H. pylori.

H. pylori NCTC 11637 strain was used. Culture mediums containing half MIC of zinc compounds (0.0005 mmol/mL for polaprezinc and 0.00125 mmol/mL for zinc acetate) were prepared. *In vitro* MICs of three antibiotics, levofloxacin (0.002 to 32 μg/mL), clarithromycin (0.016 to 256 μg/mL), and tetracycline (0.016 to 256 μg/mL), were tested using the Epsilometer test (Etest, Liofilchem, Italy). The MIC value was read at the point where the bacteriostatic ring intersects the Etest strip. The clinical breakpoints for levofloxacin, clarithromycin, and tetracycline were defined as: >1 μg/mL, >0.5 μg/mL, and >1 μg/mL, respectively, as per European Committee on Antimicrobial Susceptibility Testing Breakpoints (https://www.eucast.org/clinical_breakpoints/).

### Genome DNA extraction and analyses.

H. pylori NCTC 11637 was divided into two groups. The experimental group was incubated on medium contains 0.00125 mmol/mL zinc acetate for 3 days and the control group was incubated on blank medium for 3 days. DNA was extracted using sodium dodecyl sulfate (SDS) method (30). Qubit RNA assay kit in Qubit 2.0 Fluorometer (Life Technologies, USA) was used to quantify the extracted DNA, and its purity and integrity was determined by agarose gel electrophoresis. Eligible DNA samples were broken into fragments of about 350 bp using Covaris ultrasonic crushing instrument, and NEBNext Ultra DNA Library Prep kit for Illumina kit (NEB, USA) was used for sequencing library preparation. Library quality control was performed using Agilent 2100 Bioanalyzer (Agilent Technologies, Beijing, China), and the sequencing procedure was completed by Illumina NovaSeq PE150. Library construction, sequencing, and comparative analysis between control and experimental groups were performed by Novogene Tech (Beijing) Co., Ltd.

### RNA preparation and transcriptomic analyses.

H. pylori NCTC 11637 strain grouping was the same as Genome DNA analysis and each group were run using three replicates. Total RNA was extracted with RNAprep Pure Cell/Bacteria kit (DP430, TIANGEN, China) and quality control was assessed using agar gel electrophoresis, NanoPhotometer spectrophotometer, and Agilent 2100 bioanalyzer for integrity and purity. Eligible RNA sample was treated with Ribo-Zero Magnetic kit (Bacteria) (Epicentre, USA) to remove rRNA. The cDNA library was constructed according to the Strand-specific approach (31). For the quality of the cDNA library, Qubit2.0 Fluorometer was used for preliminary quantification, and then qRT-PCR was used for accurate quantification to ensure its effective concentration was above 2 nM. Library construction and sequencing were conducted by Novogene Tech (Beijing) Co., Ltd. Raw reads were filtered and mapped to the H. pylori NCTC 11637 genome using Bowtie2. Gene expression counts were quantified using HTSeq. Differentially expressed genes (DEGs) were analyzed using DESeq2. Genes with absolute log_2_-fold changes >0.5 and multiple *P*-value <0.05 were considered DEGs.

### Gene expression measurement by qRT-PCR.

Quantitative real-time PCR (qRT-PCR) assays were applied to detect the targeted gene expression. The same samples as in the RNA-sequencing transcriptomic analyses were used, including 3 controls incubated on blank medium and 3 incubated on medium contains 0.00125 mmol/mL zinc acetate. Based on transcriptomic analyses, 6 differentially expressed genes were selected for qRT-PCR validation. Gene-specific primers were designed using Primer Premier 5.0 and first strand cDNA was synthesized using HiFiScript cDNA Synthesis kit (Cowin Bio, China). qRT-PCRs were performed in technical triplicates using an ABI StepOne real-time PCR system (Perkin-Elmer Applied Biosystems, Foster City, CA). Reaction component and process are shown in Table S6. Gene expression and log_2_-fold changes were analyzed using the 2^-ΔΔCT^ algorithm.

### GO and KEGG pathway analyses.

First we mapped genes to Entrez Gene symbols, and then gene sets were annotated by GO (Gene Ontology) and KEGG (Kyoto Encyclopedia of Genes and Genomes) databases (32, 33). GO enrichment analysis (cellular component [CC], molecular function [MF], and biological process [BP]) and KEGG pathway enrichment analysis of DEGs were performed using clusterProfiler R package (v3.4.4), while the H. pylori 11637 reference strain annotated by Pfam (protein families database) was used as background, and Benjamini–Hochberg adjusted *P*-value <0.05 was considered significantly enriched.

### Construction of *ssrA* gene knocking-out strain.

*ssrA* gene knockout in H. pylori NCTC 11637 was constructed according to the published protocol (34) and summarized in Table S7. The plasmid PILL570, which contains a kanamycin resistance gene, was kindly provided by Yundong Sun from Shandong University, China. The recombinant plasmid for knocking out *oipA* gene (used as positive control in this study) was kindly provided by Yong Xie, Nanchang University, China. Briefly, we amplified the upstream and downstream homologous arms of *ssrA* and then inserted them into PILL570 and transformed the reconstructed plasmid into the bacteria. The targeted genes (*ssrA* and *oipA*) would be replaced by the kanamycin resistance gene.

### Knockdown of *ssrA* by siRNA.

Levofloxacin-resistant H. pylori NCTC 11637 strain with a MIC of 4–6 μg/mL induced by gradient levofloxacin exposure *in vitro* was used for analysis. SiRNA against *ssrA* was designed and synthesized by Saisofi Biotech (Jiangsu) Co., Ltd., China. The sequence is shown in Table S8. SiRNA transfection was performed following the published protocols (35). H. pylori strains were harvested from plates and washed three times with sterile 10% ice glycerol solution. One hundred μL mixture was blended with 10 μg of siRNA, and 10^9^ CFU bacteria was transferred to 0.1 cm electroporation cups (Bio-Rad, USA). Transfection was performed by electroporation using 2500V for 5 ms. At 30 min, 1 h, and 2 h after electro-transformation, bacterial RNA was extracted and reverse transcribed according to manufacturer’s protocols (Vazyme, China). The qRT-PCR components and processes are detailed in Table S9. MICs of levofloxacin to H. pylori after *ssrA* was knocked down at different time points were tested using the E-strip method.

### Data availability.

The original contributions presented in the study are publicly available. These data can be found here: https://www.ncbi.nlm.nih.gov/bioproject/PRJNA822664.
