# Supplementary material for: Transcriptomic and Functional Approaches Unveil the Role of tmRNA in Zinc Acetate Mediated Levofloxacin Sensitivity in Helicobacter pylori
Source: Microbiol Spectr. 2022 Nov 10;10(6):e01152-22. doi: 10.1128/spectrum.01152-22 (PMC9769675; doi:10.1128/spectrum.01152-22)
Supplement: Supplemental File 1 — Fig. S1 to S3. Download spectrum.01152-22-s0001.pdf, PDF file, 0.4 MB [file spectrum.01152-22-s0001.pdf]

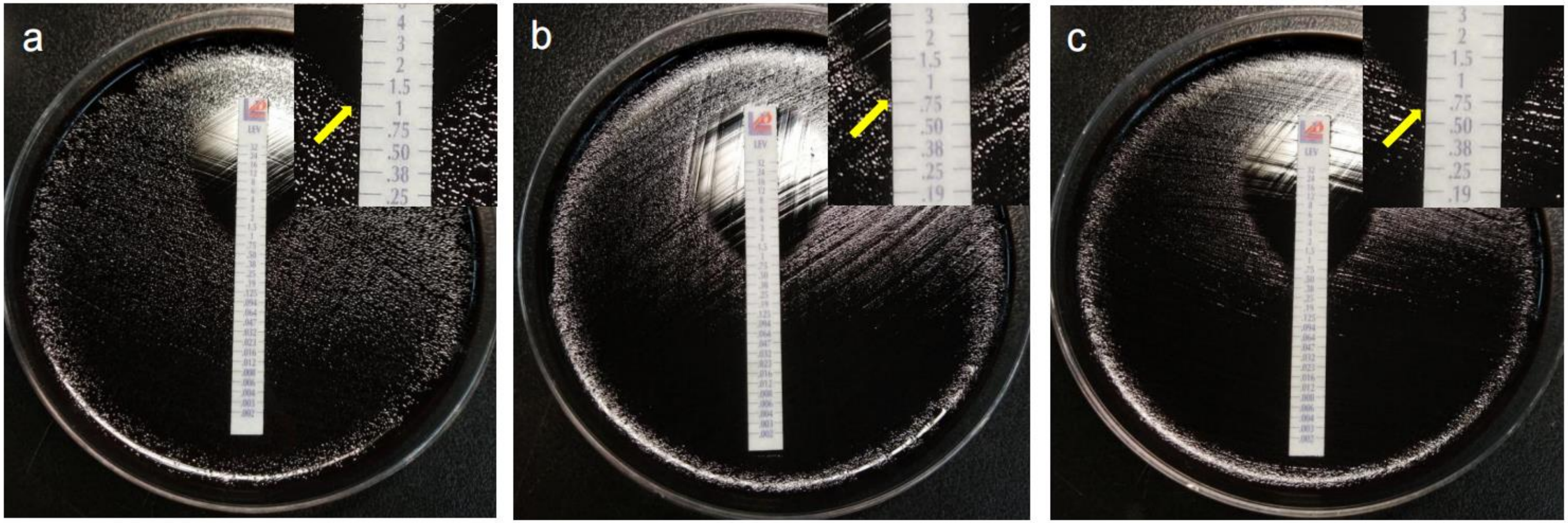

Control: 1.0-1.5 µg/ml Polaprezinc: 0.75-1.0 µg/ml Zinc acetate: 0.5-0.75 µg/ml

Supplementary Figure 1. Effects of zinc compounds on the sensitivity of *H. pylori* NCTC 11637 to levofloxacin. The MIC of levofloxacin was (a) 1.0-1.5 µg/ml in control group, (b) 0.75-1.0 µg/ml in polaprezine group, (c) 0.5-0.75 µg/ml in zinc acetate group.

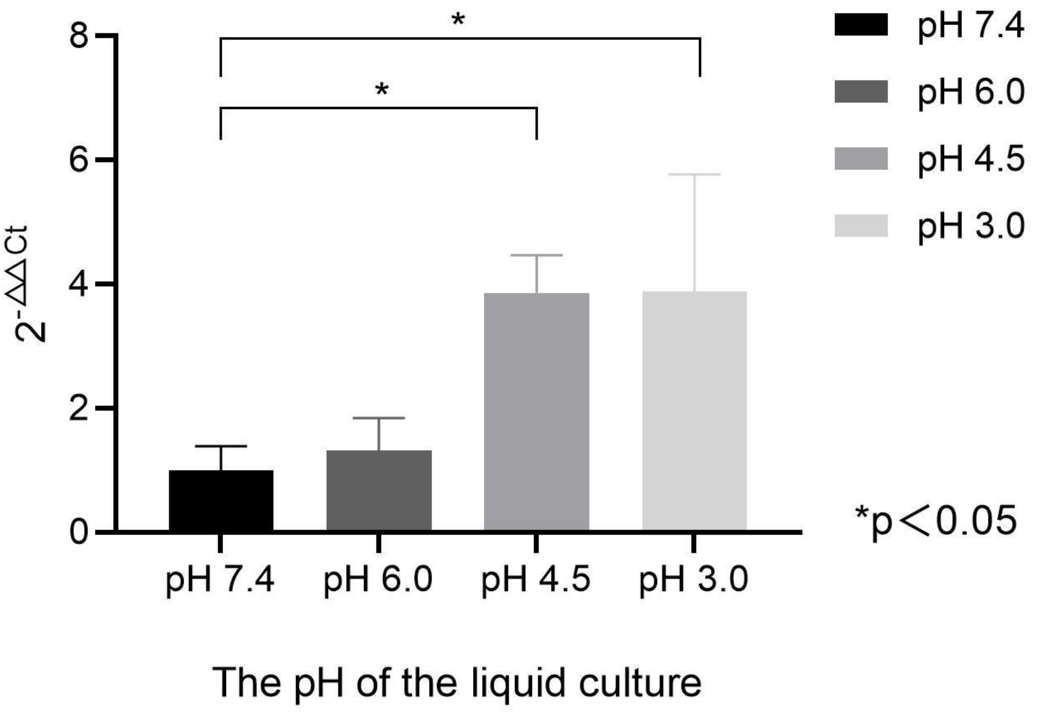

Supplementary Figure 2. Changes of *ssrA* expression in NCTC 11637 after incubated in acidity gradient liquid media evaluated by qRT-PCR. $2^{-\Delta\Delta Ct}$ was used to evaluate the difference of gene expression. Error bars indicate standard deviation.

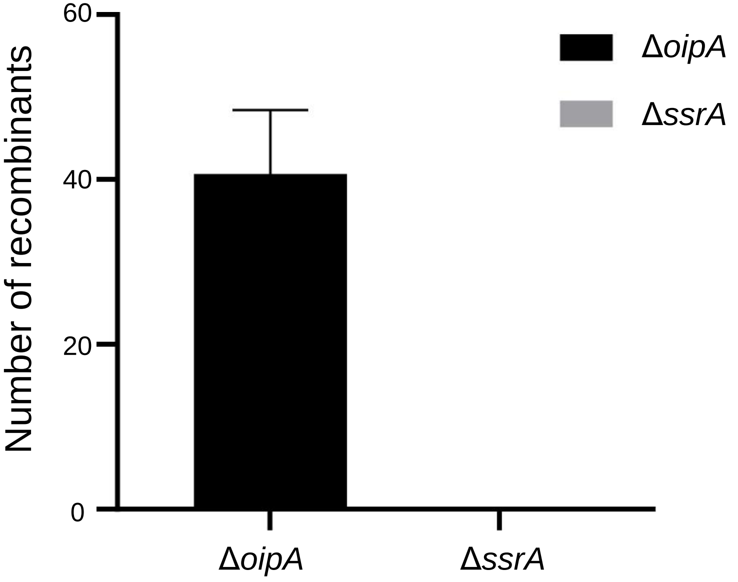

Supplementary Figure 3. The number of recombinants obtained for NCTC 11637 following introduction of the plasmids that knock out *ssrA* and *oipA*. Δ*oipA* was used as a positive control. Recombinants were the colonies obtained after transformation and selected on kanamycin-contained plates. The vertical axis corresponds to the number of recombinants.
